# Supplementary figures and images for: Phytochemical content, especially spermidine derivatives, presenting antioxidant and antilipoxygenase activities in Thai bee pollens
Source: PeerJ. 2022 May 25;10:e13506. doi: 10.7717/peerj.13506 (PMC9147323; doi:10.7717/peerj.13506)

(A)

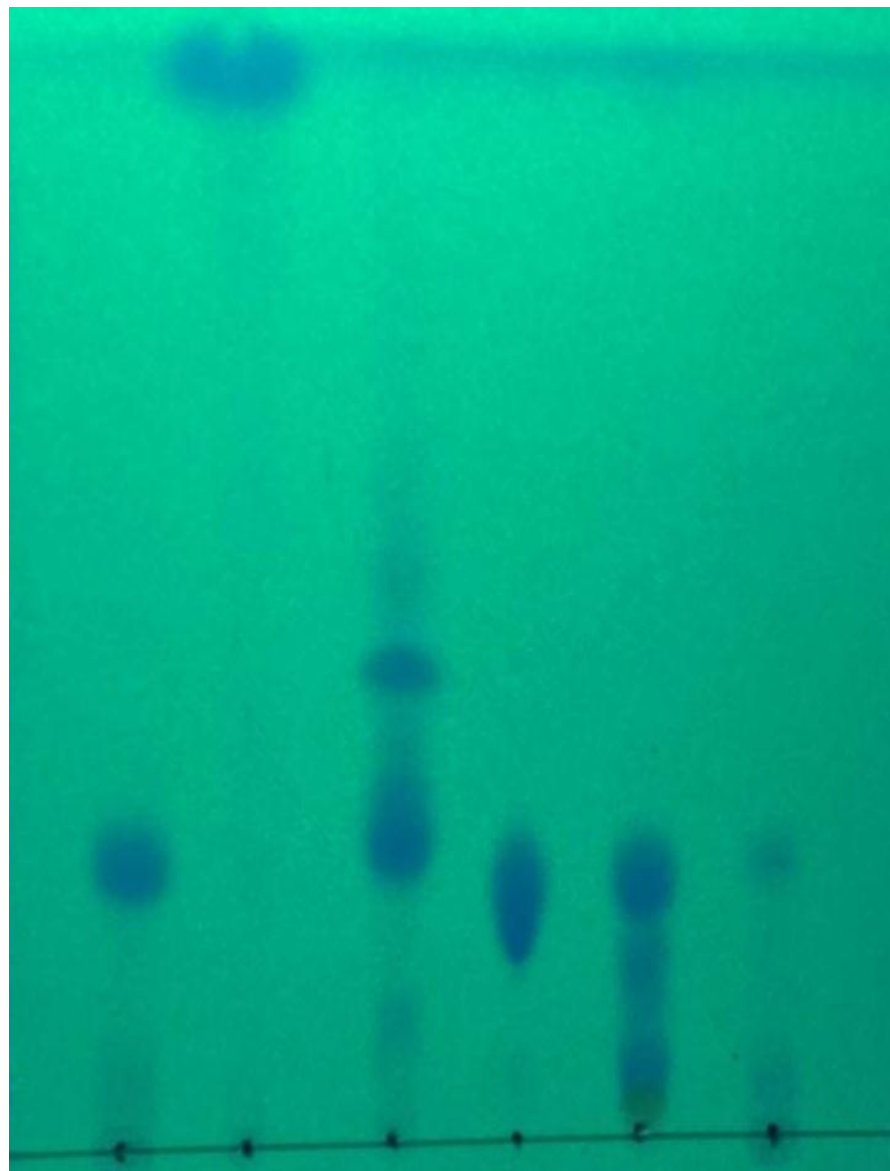

(B)

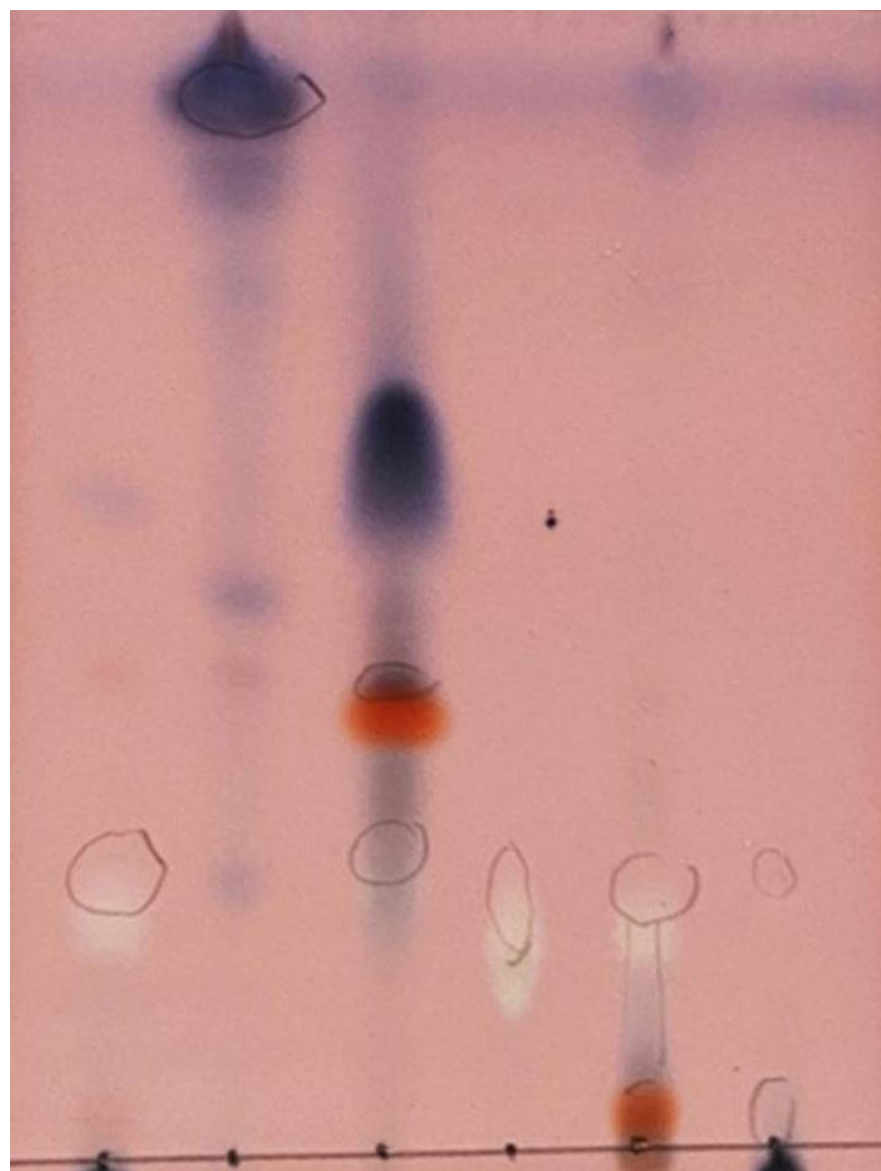

Supplement: Supplemental Information 2 — The mobile phase was 7% MeOH-DCM. [file peerj-10-13506-s002.pdf]

Compound 1

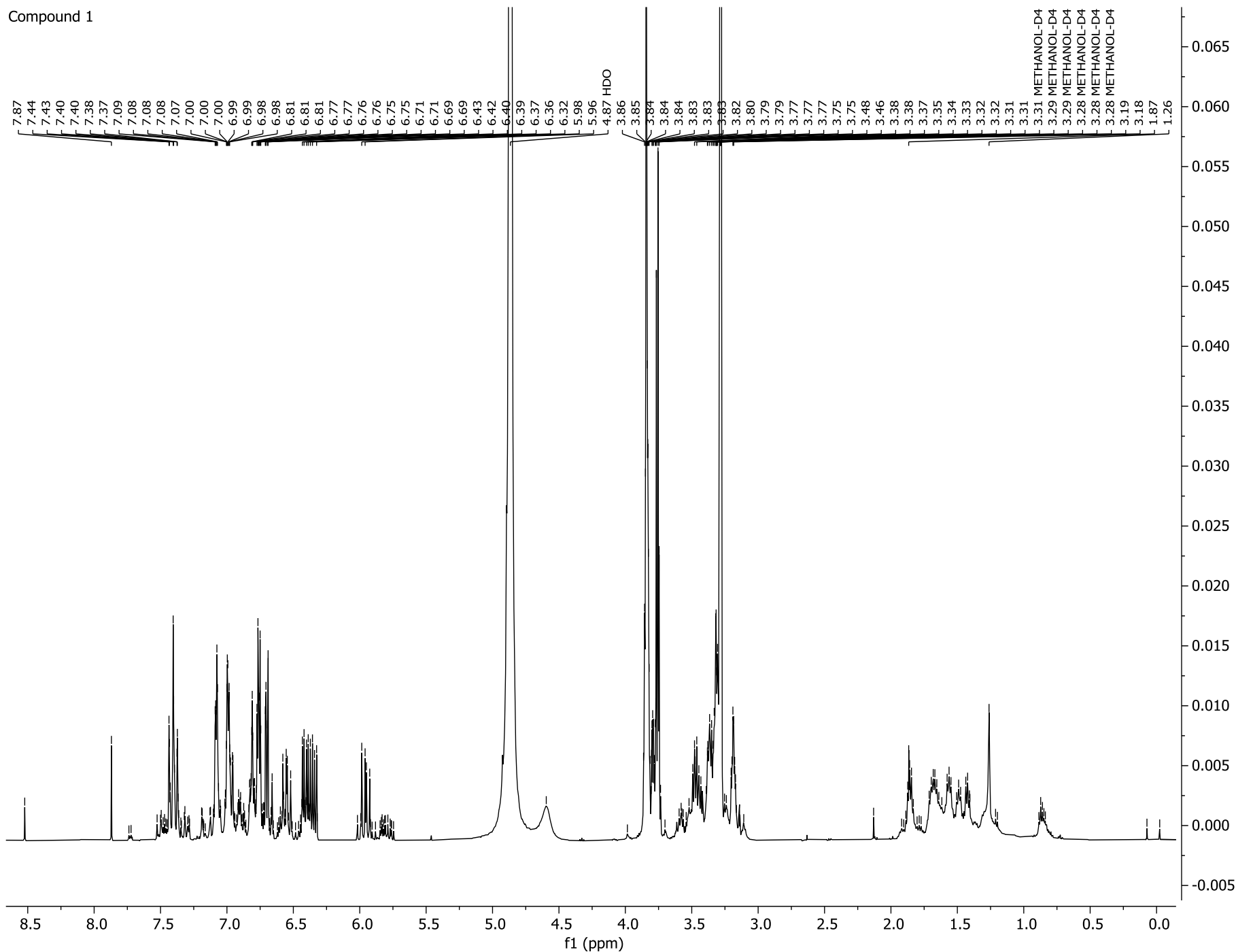

Supplement: Supplemental Information 3 [file peerj-10-13506-s003.pdf]

Compound 2

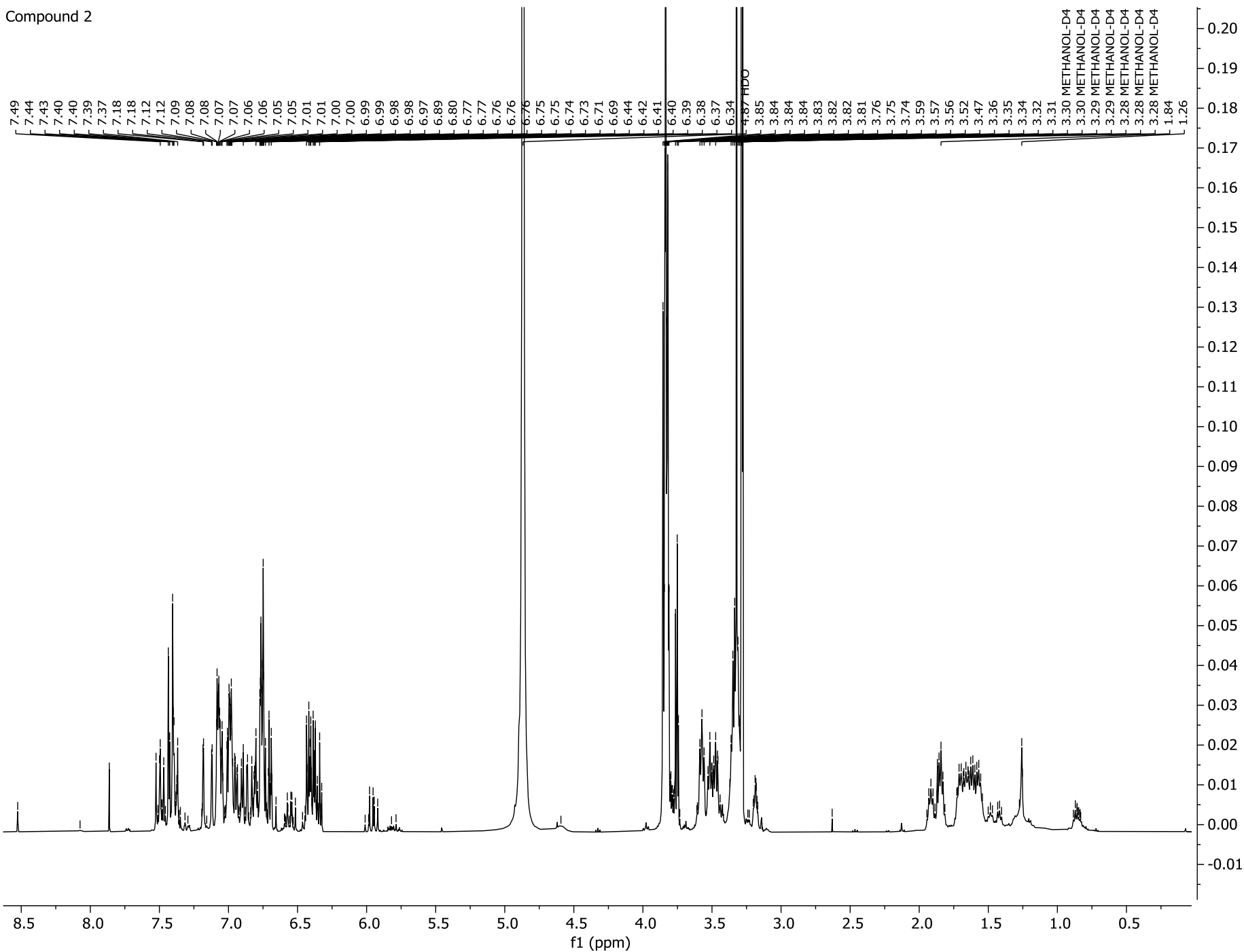

Supplement: Supplemental Information 4 [file peerj-10-13506-s004.pdf]

Comment 1 MimosaF3-1(2)

Comment 2

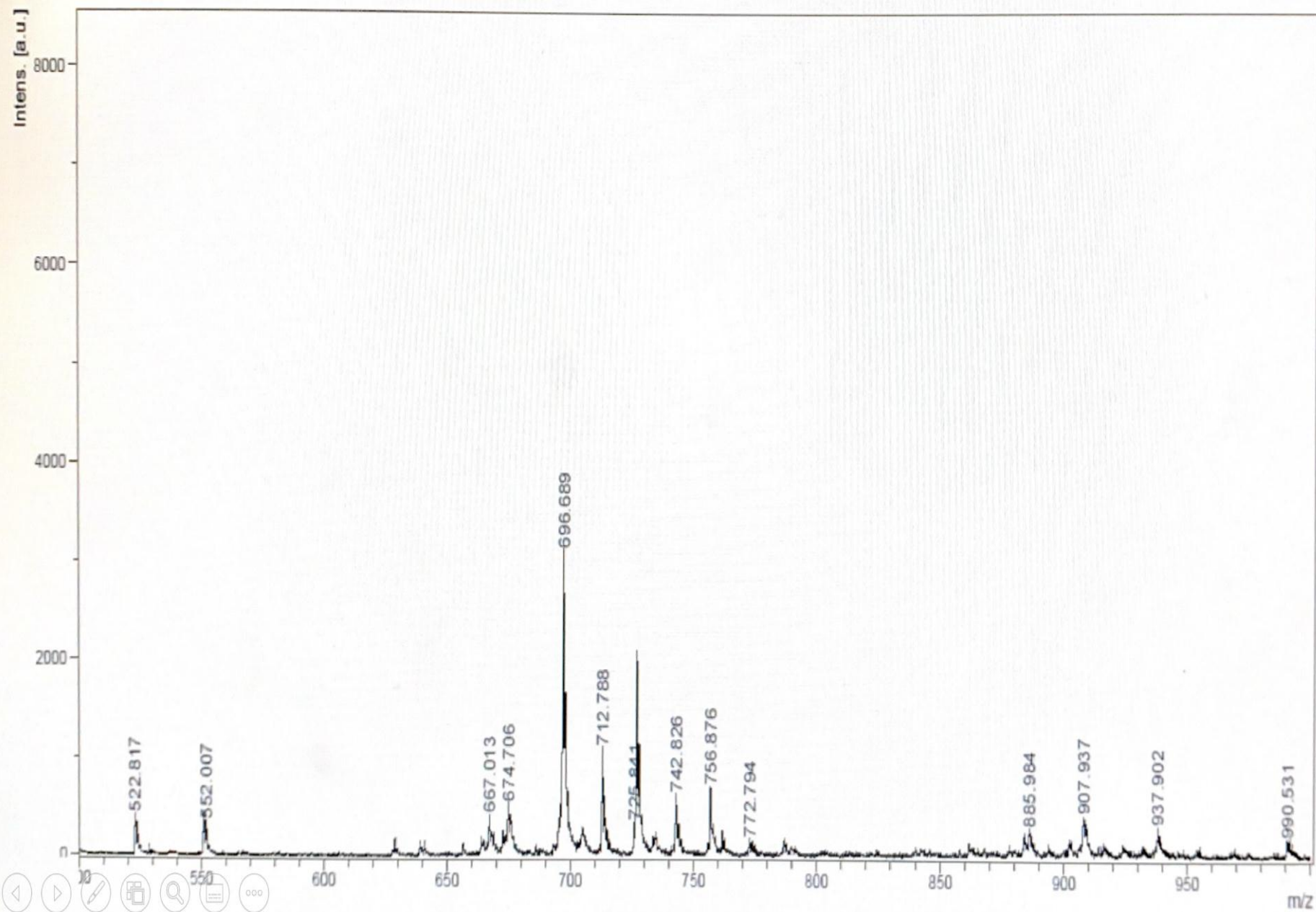

Supplement: Supplemental Information 6 [file peerj-10-13506-s006.pdf]

Comment 1 MimosaF3-2(3)

Comment 2

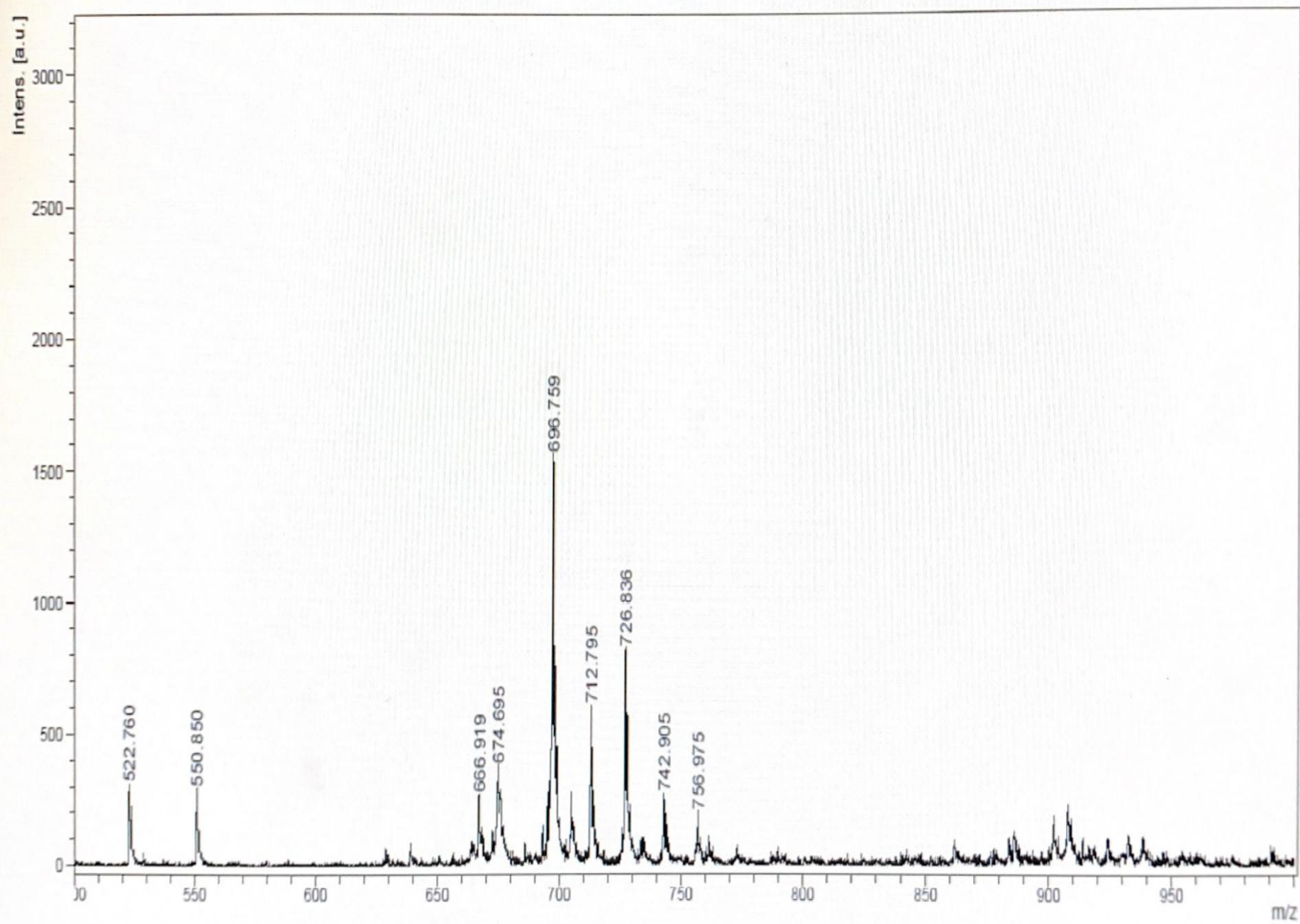

Supplement: Supplemental Information 7 [file peerj-10-13506-s007.pdf]
